# Chronic hepatitis B virus infection deteriorates disease outcome of coronavirus disease 2019 in hamster

**DOI:** 10.1002/mco2.499

**Published:** 2024-02-28

**Authors:** Lunzhi Yuan, Xuan Liu, Yi Guan, Tong Cheng, Ningshao Xia

**Affiliations:** ^1^ State Key Laboratory of Vaccines for Infectious Diseases, National Institute of Diagnostics and Vaccine Development in Infectious Diseases, School of Life Sciences & School of Public Health Xiamen University Xiamen Fujian China; ^2^ Clinical Center for Bio‐Therapy, Zhongshan Hospital Fudan University (Xiamen Branch) Xiamen Fujian China; ^3^ State Key Laboratory of Emerging Infectious Diseases, School of Public Health, Li Ka Shing Faculty of Medicine The University of Hong Kong Hong Kong SAR China

Dear Editor,

The pandemic of severe acute respiratory syndrome coronavirus 2 (SARS‐CoV‐2) is widely recognized as a global public health crisis. Over the past three years, SARS‐CoV‐2 has been mutating and resulting in varying degrees of coronavirus disease 2019 (COVID‐19), characterized by symptoms such as fever, cough, weakness, severe pneumonia, and even death. After the pandemic waves of the Delta and Omicron sublineages, the XBB variants of SARS‐CoV‐2 are still emerging in local areas. It has been reported that patients with chronic liver diseases, including those with chronic hepatitis B virus (HBV) infection,^1^ are at a higher risk of experiencing poor outcomes from COVID‐19. However, due to the lack of clinical samples and appropriate animal models, our understanding of various aspects of SARS‐CoV‐2 infection and the progression of COVID‐19 in patients with chronic HBV infection (CHB) remains incomplete. Therefore, in this study, we aimed to replicate the dual infection of HBV and SARS‐CoV‐2 in a sensitive rodent model, namely the hamster. We infected 4‐week‐old male hamsters with AAV‐HBV (1×10^9^ vg per hamster) to establish persistent viremia of HBV surface antigen (HBsAg) (Figure S1).

Subsequently, we mimicked different stages of chronic HBV infection in patients by intranasally infecting the hamsters with 1×10^4^ PFU of the SARS‐CoV‐2 Delta variant at 4 weeks (4 W) and 16 weeks (16 W) after HBV infection. The control group consisted of hamsters infected solely with SARS‐CoV‐2. Interestingly, among the hamsters, six out of 12 control hamsters, one out of 12 HBV 4 W hamsters, and 10 out of 12 HBV 16 W hamsters died within 14 days post‐infection (dpi) with SARS‐CoV‐2 (Figure 1A). Furthermore, at 7 dpi, the control, HBV 4 W, and HBV 16 W hamsters exhibited body weight losses of 15.13 ± 2.59%, 9.45 ± 2.47%, and 20.41 ± 3.18%, respectively (Figure 1B). These findings suggest that HBV 4 W and HBV 16 W hamsters developed contrary disease outcomes following SARS‐CoV‐2 infection.

**FIGURE 1:**
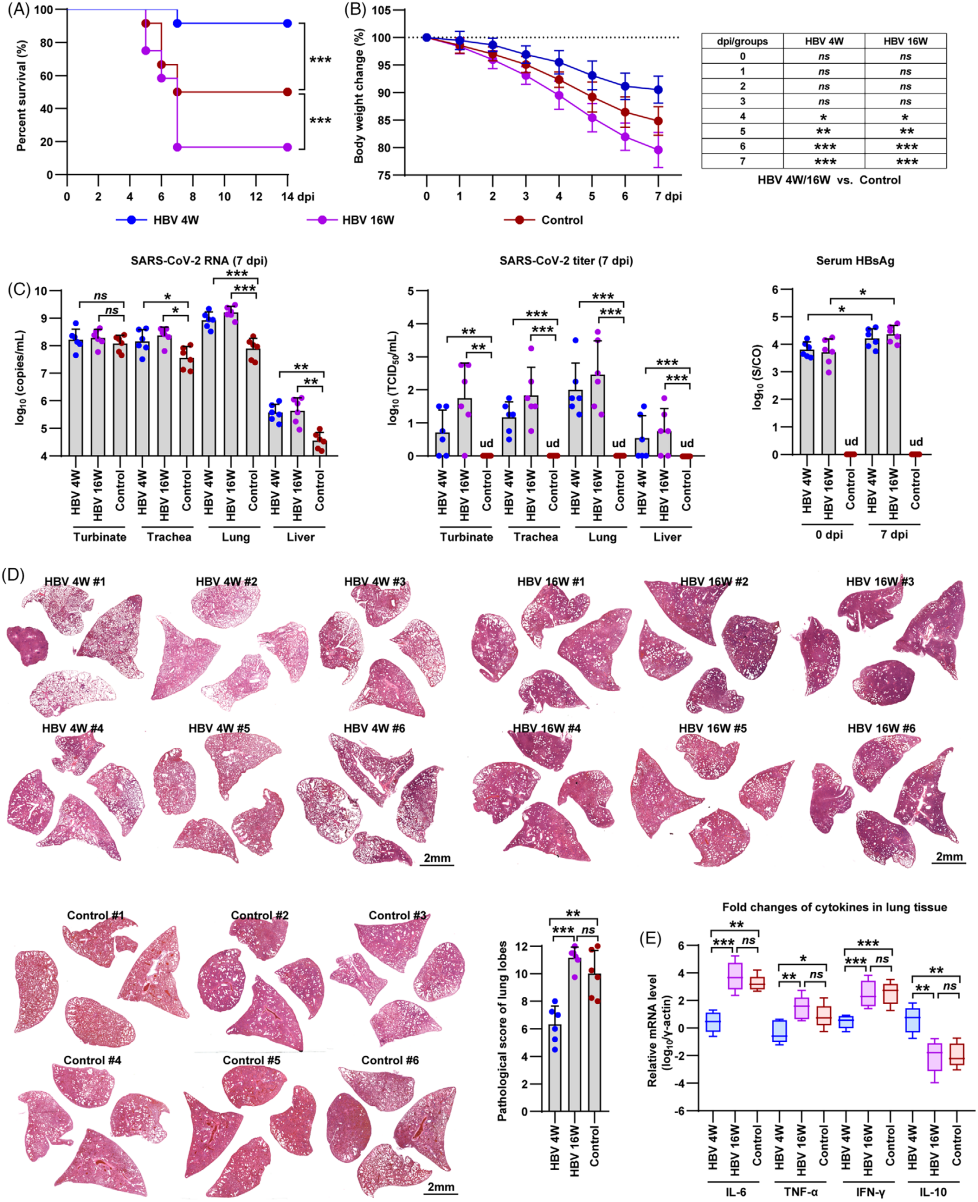
Severe acute respiratory syndrome coronavirus 2 (SARS‐CoV‐2) infection in hepatitis B virus (HBV) hamster model. (A) The survival rate of the SARS‐CoV‐2‐infected hamsters was from 0 to 14 dpi (n = 12). Significance was calculated using the Long‐rank test. (B) Record of body weight changes of the hamsters from 0 to 7 dpi (n = 6). Significance was calculated using a two‐way analysis of variance (ANOVA). (C) Viral load of SARS‐CoV‐2 and HBV in the hamster model. Left panel, the levels of SARS‐CoV‐2 RNA in turbinate, trachea, lung, and liver tissues collected at 7 dpi were measured by reverse transcription‐polymerase chain reaction (RT‐PCR), using primers to amplify the SARS‐CoV‐2 ORF1ab gene (*n* = 6). Middle panel, virus titer in these samples was calculated by 50% tissue culture infective dose (TCID_50_) in a 96‐well plate (*n* = 6). Right panel, the levels of HBsAg in hamster serum collected at 0 and 7 dpi were measured by enzyme‐linked immunosorbent assay (ELISA) (*n* = 6). (D) Screened images of H&E staining of the lung lobes collected from the HBV 4 W (up‐left), HBV 16 W (up‐right), and control hamsters (down‐left) sacrificed at 7 dpi, respectively (Bar = 2 mm). Comprehensive pathological scores for lung sections were determined based on the severity and percentage of injured areas of each lung lobe. For each group, approximately 24 lung lobes were collected from six individual hamsters and were scored (Table. S1). (E) Fold changes for mRNA levels of representative cytokines in the hamster lung tissues collected at 7 dpi were measured by RT‐PCR (*n* = 6). The mRNA levels of cytokines were standardized to the housekeeping gene γ‐actin. (C, E) Significance was calculated using one‐way ANOVA. Data are presented as the means ± SD. Two‐sided *p*‐values < 0.01 were considered significant: *p < 0.01, **p < 0.001, ***p < 0.0001, ns indicates no significance, ud indicates undeletable.

We proceeded to examine the presence of SARS‐CoV‐2 RNA and its titer in the nasal turbinate, trachea, lung, and liver of the hamsters sacrificed at 7 days post‐infection (dpi). The results obtained through RT‐PCR revealed higher levels of SARS‐CoV‐2 RNA in both the HBV 4 W and HBV 16 W hamsters compared to the control hamsters at 7 dpi (Figure 1C, left). Additionally, titration results indicated the absence of detectable infectious SARS‐CoV‐2 particles in the nasal turbinate, trachea, lung, and liver of the control hamsters at 7 dpi, while a significant viral titer was observed in more than 50% of the HBV 4 W and HBV 16 W hamsters at the same time point (Figure 1C, middle). Notably, there was a substantial variation in both SARS‐CoV‐2 RNA levels and titers among the tissues collected from the HBV 4 W and HBV 16 W hamster groups (Figure 1C). Additionally, SARS‐CoV‐2 infection caused a slight increase in serum HBsAg levels in both the HBV 4 W and HBV 16 W hamsters (Figure 1C, right). Interestingly, both the 4 and 16 W HBV hamsters showed diffuse expression of HBsAg in the liver tissues and delayed clearance of SARS‐CoV‐2 N protein in the lung tissues collected at 7 dpi, respectively (Figure S2).

In addition, we investigated the pathological changes in the lung tissues of hamsters collected at 7 days post‐infection (dpi) as previously described.^2^ Based on the results of hematoxylin and eosin (H&E) staining of fixed lung lobes, the HBV 16 W hamsters exhibited more severe lung injury compared to the control hamsters, while the HBV 4 W hamsters exhibited milder lung injury than the other two groups (Figure 1D). In addition, we quantified the severity of lung pathology using a comprehensive pathological scoring system that included factors such as alveolar septal thickening and consolidation, hemorrhage, exudation, pulmonary edema and mucus, and inflammatory cell recruitment and infiltration in all lobes of the hamster lungs. The control, HBV 4 W, and HBV 16 W hamsters had mean comprehensive pathologic scores of 10.00 ± 1.69, 6.33 ± 1.32, and 11.17 ± 0.77, respectively (Figure 1D and Table S1). Furthermore, we evaluated the relative mRNA levels of representative cytokines in the lung and liver tissues collected at 7 dpi. Notably, the HBV 4 W hamsters exhibited a significant decrease in proinflammatory cytokines, including interleukin 6 (IL‐6), tumor necrosis factor‐alpha (TNF‐α), and interferon‐gamma (IFN‐γ), and an increase in the anti‐inflammatory cytokine IL‐10 (Figure 1E, Figure S3, and Table S2). In contrast, HBV 16 W hamsters did not show significant changes in the mRNA levels of these cytokines compared to the control group (Figure 1E).

In conclusion, the presence of persistent HBsAg viremia delays viral clearance of SARS‐CoV‐2 in the hamster model. The results of survival rate, body weight changes, pathological changes, and cytokine mRNA levels in lung tissues indicate that long‐term persistence of HBsAg viremia (HBV 16 W) is likely to worsen the disease outcome of COVID‐19 in the hamster model, whereas short‐term persistence of HBsAg viremia (HBV 4 W) may ameliorate the disease severity. Chronic hepatitis B (CHB) typically disrupts innate and adaptive immune responses, and the immunopathophysiological process is complex.^3^ Based on the immunological characteristics of CHB and COVID‐19 patients and,^4^, ^5^ we hypothesized that an exaggerated innate immune response and an inflammatory immune microenvironment in the later stages of CHB contribute to poor disease outcome after SARS‐CoV‐2 co‐infection. Conversely, the early stages of CHB are characterized by immunosuppression, which may result in an inadequate innate immune response and the production of proinflammatory cytokines. Therefore, targeting the abnormal immune response and organ failure are key steps to reserve the complicated pathology progress of SARS‐CoV‐2 co‐infection in CHB patients. For example, intravenous injection of mesenchymal stem cells has been demonstrated available to down‐regulate the severity of liver failure, pneumonia, and cytokine storm. Meanwhile, a combined antiviral treatment might enhance the therapeutic effect of immunotherapy. Overall, Further animal studies and analysis of clinical specimens are needed to validate our hypothesis.

To summarize, this study provides a highly representative animal model of SARS‐CoV‐2 infection in CHB patients and highlights the long‐term persistence of HBsAg viremia as a risk factor. These findings may provide valuable insights into understanding the immunopathophysiological mechanisms underlying HBV/SARS‐CoV‐2 co‐infection and inform clinical treatment strategies.

## AUTHOR CONTRIBUTIONS

Y.L.Z. and L.X. contributed equally to this study. Y.L.Z. and L.X. performed the cell and animal experiments. Y.L.Z. and L.X. wrote the manuscript. G.Y., C.T., and N.S.X. supervised the study. All authors have read and approved the final manuscript.

## CONFLICT OF INTEREST STATEMENT

The authors declare no conflict of interest.

## FUNDING INFORMATION

This work was supported by grants from the National Natural Science Foundation of China (82002139; 32201152), Funding support from the Guangdong Government (HZQB‐KCZYZ‐2021014; 190824215544727), Shenzhen Science and Technology Program (JCYJ20220530143407016).

## ETHICS STATEMENT

In this study, the virus and animal studies were approved by the ethics committee of the Guangdong‐Hong Kong Joint Laboratory of Emerging Infectious Diseases/Joint Laboratory for International Collaboration in Virology and Emerging Infectious Diseases (Key Laboratory of Ministry of Education), Joint Institute of Virology (Shantou University/The University of Hong Kong) (Approval number: SUMC2022‐051).

## Supporting information

Supporting information

## Data Availability

The information on supplemental tables, methods, and materials is shown in the Supporting Information file. Further information and requests for resources and reagents should be directed to and will be fulfilled by Dr. Lunzhi Yuan (yuanlunzhi@xmu.edu.cn; ORCID: 0000‐0001‐7480‐0287).
