# Supplementary material for: Chronic hepatitis B virus infection deteriorates disease outcome of coronavirus disease 2019 in hamster
Source: MedComm (2020). 2024 Feb 28;5(3):e499. doi: 10.1002/mco2.499 (PMC10901280; doi:10.1002/mco2.499)
Supplement: Supplementary file 1 — Supporting information [file MCO2-5-e499-s001.docx]

Supplementary Information

**Chronic HBV infection deteriorates disease outcome of COVID-19 in hamster**

Lunzhi Yuan^1^*^#^, Xuan Liu^2#^, Yi Guan^3^, Tong Cheng^1^*, Ningshao Xia^1^*

^1^State Key Laboratory of Vaccines for Infectious Diseases, National Institute of Diagnostics and Vaccine Development in Infectious Diseases, School of Life Sciences & School of Public Health, Xiamen University, Xiamen, Fujian, China

^2^Clinical Center for Bio-Therapy, Zhongshan Hospital, Fudan University (Xiamen Branch), Xiamen, Fujian, China

^3^State Key Laboratory of Emerging Infectious Diseases, School of Public Health, Li Ka Shing Faculty of Medicine, The University of Hong Kong, Hong Kong SAR, China

**Materials and Methods**

**Experimental Animal and Biosafety**

Six- to eight-week-old male hamsters (LVG strain) were used in this study. The Syrian Hamster was raised in the specific pathogen free animal feeding facilities. All the animal experiments were approved by the Medical Ethics Committee (SUMC2022-051). All experiments with infectious SARS-CoV-2 were performed in the biosafety level 3 (BSL-3) and animal biosafety level 3 (ABSL-3) facilities. Our staff wore powered air-purifying respirators that filtered the air, and disposable coveralls when they cultured the virus and handled animals that were in isolators. The researchers were disinfected before they left the room and then showered on exiting the facility. All facilities, procedures, training records, safety drills, and inventory records were subject to periodic inspections and ongoing oversight by the institutional biosafety officers who consult frequently with the facility managers.

**Virus Stock of SARS-CoV-2**

The SARS-CoV-2 Omicron Delta variant AP-1914 (share an identical sequence with EPI_ISL_2385091) was passaged on Vero cells (#CCL-81, ATCC). Viral stocks were prepared in Vero cells with DMEM containing 2% FBS, 5ug/mL TPCK-trypsin, 1% Penicillin-Streptomycin and 30mmol/L MgCl_2_. Viruses were harvested and stored in ultra-low temperature freezer. The titers were determined by means of plaque assay in Vero cells.

**Establishment of HBV/SARS-CoV-2 hamster model**

To simulate chronic HBV infection in human patients, four-week male hamsters were anesthetized by isoflurane (#R510-22, RWD Life Science) and intraperitoneally injected with 1×10^9^ vg of AAV-HBV (#AMV-001, Beijing Five Plus Gene Technology) diluted in 500μL of PBS (#10010031, GIBCO). To simulate SARS-CoV-2 infection in human patients, mock hamsters and hamsters at 4-week or 16-week after injection of AAV-HBV were anesthetized by isoflurane and intranasally inoculated with 1×10^4^ PFU of SARS-CoV-2 diluted in 200μL of PBS. Body weight of these hamsters were measured by an electronic balance. Hamsters were euthanized at the indicated time point for detection of viral load, changes of cytokines as well as pathological examination.

**Detection of SARS-CoV-2 and HBV**

For the solid organ samples, we collect 1mg turbinate, 0.1 mg trachea, 0.1 mg lung and 0.1 mg liver in 1mL PBS for homogenate and detection of viral RNA and viral titer. Viral RNA was extracted by using a QIAamp Viral RNA Mini kit (#52906, Qiagen) according to the manufacturer's instructions. The RT-PCR was conducted by using the SLAN-96S Real-Time System (Hongshi, Shanghai, China) with a SARS-CoV-2 RT-PCR Kit from Wantai (Beijing, China). Relative Viral RNA of SARS-CoV-2 ORF1ab gene was determined using primer pairs and probes provided in the kit. Viral RNA copies were expressed on a log10 scale after normalized to the standard curve obtained by using ten-fold dilutions of a SARS-CoV-2 stock.

The titers of homogenized tissues were measured by plaque assay and half tissue culture infective dose (TCID_50_) titration method in Vero cells seeded in 96-well plates. In the TCID_50_ titration assay, Vero cells were incubated with 100μL of original tissue homogenates and 10-fold serial diluted samples for one hour. And then, we renewed fresh medium and observed cytopathic effect (CPE) at three days after incubation. We defined that all cells without cytopathic effect indicate “zero”. The serum neutralization titers were measured by a titration method based on TCID_50_ inhibition. We added 10μL of serum sample in 90μL of medium for each well and performed 2-fold gradient dilution. After that, 100μL of serum sample and 100 TCID_50_ virus in 100μL medium were co-incubated with Vero cells for one hour. Finally, we renewed fresh medium and observed inhibition of CPE at three days after co-incubation. The levels of HBsAg and HBsAb in serum were detected by ELISA kits provided by Beijing Wantai.

**Detection of Cytokine in mRNA levels**

The lung tissues were cleaved into small pieces and soaked in RNAlater (#AM7021, Invitrogen). Total RNAs in lysed lung tissues were extracted with RNeasy Mini kit (#74106, Qiagen) and reverse-transcribed to cDNA with Fast-King Strand cDNA Synthesis Kit (#FP313, TIANGEN, Beijing). Diluted cDNAs (1:10) were quantified using SYBR Green I-based real-time PCR using the LightCycler® 480 instrument (Roche) per manufacturer’s instructions. Threshold cycle (Ct) of each gene was normalized to the internal reference gene (hamster γ-actin) and comparative Ct (2-ΔΔCt) method was utilized to calculate changes in chemokine and cytokine gene expression profile. The detailed information of the primers was shown in Table S2.

**Histopathological Studies**

For pathological analysis, lung tissues were fixed in formalin for more than 72 hours, dehydrated and then embedded in paraffin wax. The wax block of lung tissues was cut into 4μm sections for pathological staining and analysis. H&E staining was employed for analysis of general lung pathogenic lesions including pulmonary edema, consolidation and inflammation. The standards for pathological score of lung tissues in this study are derived from our previous study in hamster model. Comprehensive pathological scoring of lung sections was performed according to the degree of lung lesions including alveolar septum hyperplasia, consolidation and impairment of alveolar structure, fluid exudation, mucus suppository, thrombus, inflammation recruitment and infiltration of immune cells in each individual lung lobe. For each hamster, three or four lung lobes were employed for evaluation of comprehensive pathological score. In brief, H&E staining result of each lung lobe was analyzed for its severity of pathological change. The pathological score included: a) Alveolar septum thickening and consolidation; b) Hemorrhage, exudation, pulmonary edema and mucous; c) Recruitment and infiltration of inflammatory immune cells. For each issue, scores were related to the severity: 0 indicated no pathological change was observed, 1 indicated moderate pathological change, 2 indicated mild pathological change, 3 indicated severe pathological change and 4 indicated very severe pathological change. In conclusion, scores of such three issues were added as the comprehensive pathological score of a lung lobe, and the average comprehensive pathological score of the lobes indicated the severity of lung pathology in an evaluated hamster. The pathological reagents include immunohistochemistry kit (#KIT-9730), Hematoxylin (#CTS-1096) and Eosin (#CTS-4094) were purchased from Maxim Biotechnology (Fuzhou, China). The antibodies for immunohistochemistry staining of HBsAg (15A7-1) and SARS-CoV-2 N protein (7G11) were house-keeping. The images of whole lung lobes were screened by a high-throughput screening microscope system (EVOS M7000, Invitrogen of Thermo-Fisher Scientific).

**Animal and Sample size justification**

Sample sizes maximized considering limits in BSL-3 working capacity, numbers of animals that can be handled under ABSL-3 conditions and availability of well-trained staffs.

**Statistical Analysis**

Student’s unpaired two-tailed t-test, one-way ANOVA, two-way ANOVA and Long-rank test were performed using GraphPad Prism 8.0 (GraphPad Software). Data are presented as the means ± SD. Two-sided p-values <0.01 were considered significant: *P <0.01, **P <0.001, ***P <0.0001, ns indicates no significance, ud indicates undetectable.


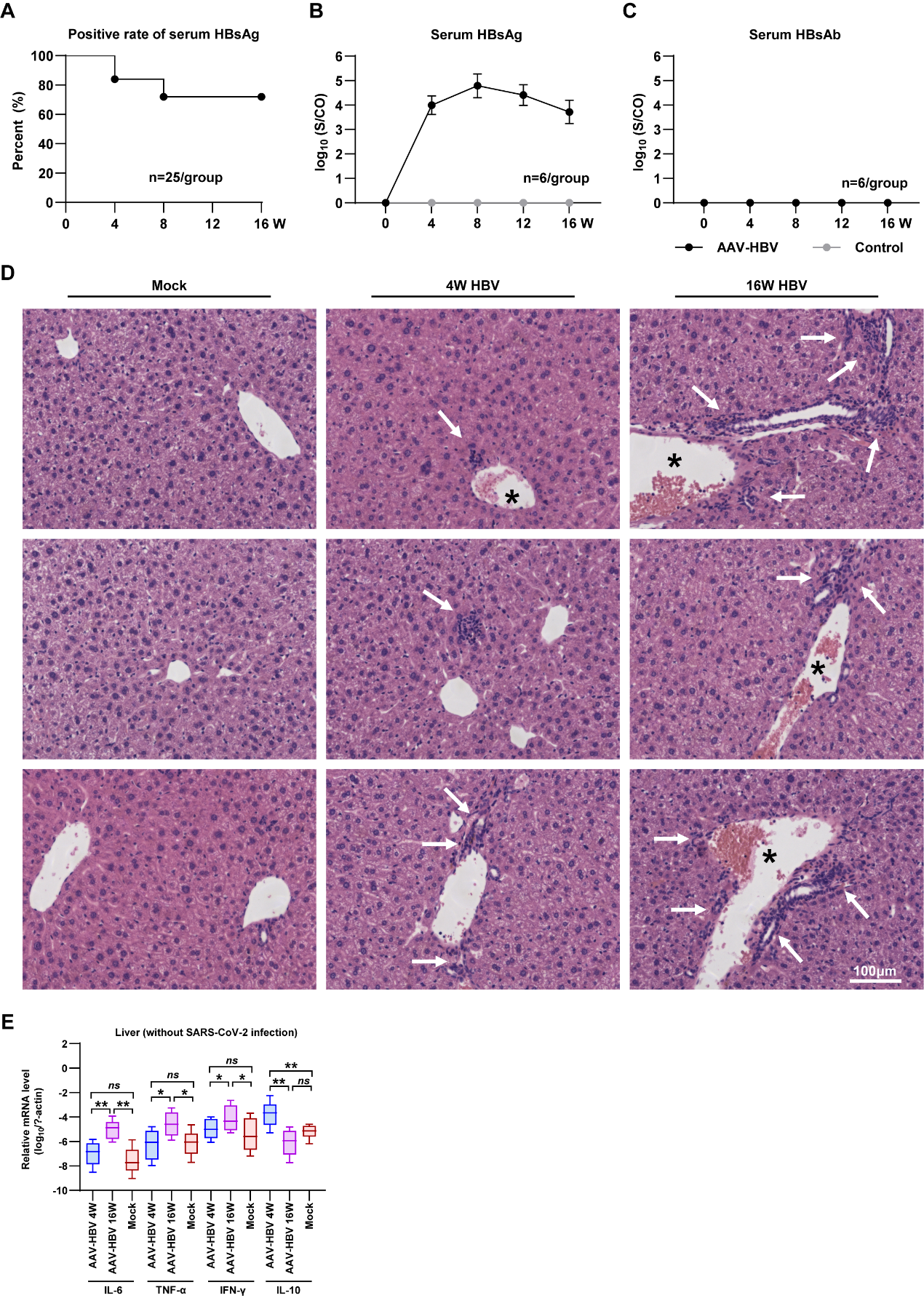


**Figure S1. Characteristics of AAV-HBV hamster model. (A)** Positive rate for serum HBsAg in the hamsters with AAV-HBV infection. 18 out of 25 hamsters were positive of HBsAg for over 16 weeks. The levels of **(B)** HBsAg and **(C)** anti-HBsAg (HBsAb) in serum samples of hamsters with or without AAV-HBV infection were detected by ELISA. HBsAg was detectable only in the hamsters with AAV-HBV infection. HBsAb was undetectable in both the AAV-HBV hamsters and those without AAV-HBV infection throughout the course. **(D)** H&E staining for the liver tissues collected the hamsters without AAV-HBV infection (mock), hamsters with AAV-HBV infection for 4 weeks (4W HBV) and 16 weeks (16W HBV) were shown (Bar=100μm). Infiltration of inflammatory immune cells (white arrow) and hemorrhage (black asterisk) were observed in the liver tissues of 16W HBV hamsters. **(J)** Fold changes for mRNA levels of representative cytokines in the liver tissues of these hamsters were measured by RT-PCR (n=6). The mRNA levels of cytokines were standardized to the house-keeping gene γ-actin. Representative inflammatory cytokine genes include IL-6, TNF-α and IFN-γ. IL-10 is a typical anti-inflammatory cytokine gene.


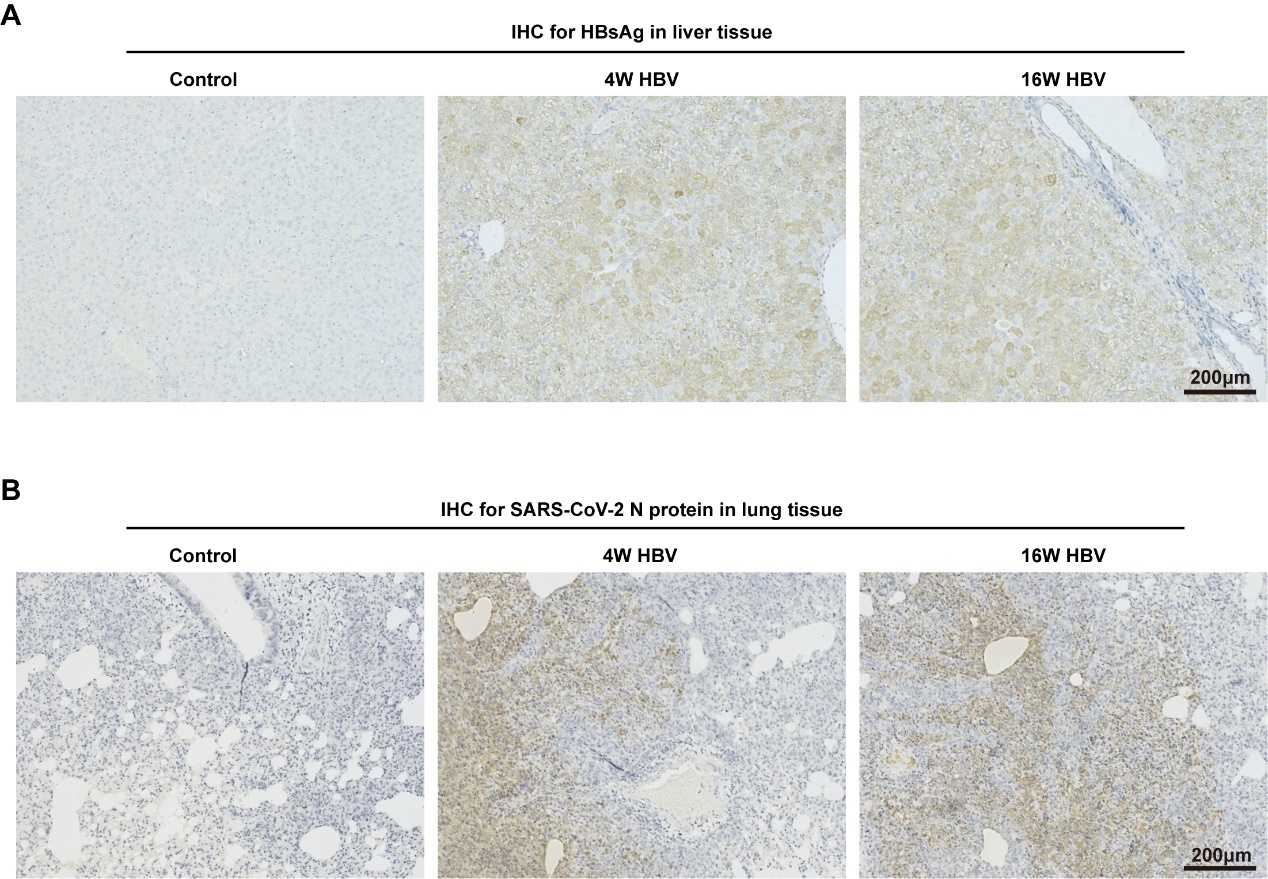


**Figure S2. Immunohistochemistry staining for HBsAg in liver tissue section and SARS-CoV-2 N protein in lung tissue section.** Liver and lung tissues were collected and fixed from the non-HBV hamsters (control group), 4W HBV and 16W HBV hamsters at 7 dpi of SARS-CoV-2, respectively (Bar=200μm). **(A)** Diffuse distribution of HBsAg positive cells were detectable in the liver tissues of 4W HBV and 16W HBV hamsters, which indicates persistent HBV replication. **(B)** Diffuse distribution of SARS-CoV-2 N protein positive cells were detectable in the lung tissues of 4W HBV and 16W HBV hamsters, which indicates delay of SARS-CoV-2 clearance.


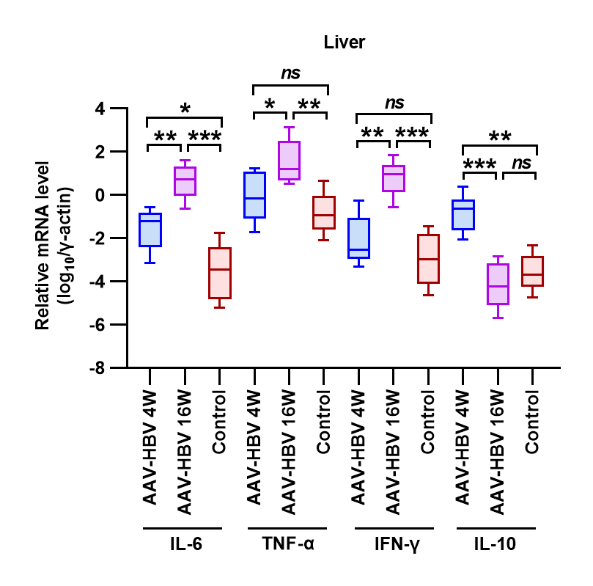


**Figure S3. Detection of representative cytokines in liver tissues at 7 dpi of SARS-CoV-2.** Fold changes for mRNA levels of IL-6, TNF-α, IFN-γ IL-10 in the hamster liver tissues collected at 7 dpi were measured by RT-PCR (n=6). The mRNA levels of cytokines were standardized to the house-keeping gene γ-actin.

**Table S1. Comprehensive pathological score of the hamster lung lobes collected at 7 dpi.**

| **Group** | **No.** | **Pathological lesions** | | | **Comprehensive pathological score** | **Average pathological score** |  |
| --- | --- | --- | --- | --- | --- | --- | --- |
|  |  |  |  |  |  |  |  |
|  |  | **Alveolar septum hyperplasia and consolidation** | **Pulmonary edema, hemorrhage and mucus suppository** | **Recruitment and infiltration of inflammatory cells** |  |  |  |
|  |  |  |  |  |  |  |  |
| **AAV-HBV 4W** | **#1** | 4+1+1+0 | 4+1+1+1 | 4+1+0+0 | 12+3+2+1 | 4.5 |  |
|  | **#2** | 3+2+2+2 | 3+2+2+2 | 2+2+1+1 | 8+6+5+5 | 6 |  |
|  | **#3** | 3+3+2+1 | 3+3+3+2 | 3+3+2+1 | 9+9+7+4 | 7.25 |  |
|  | **#4** | 4+3+2+1 | 4+3+3+1 | 4+3+3+1 | 12+9+8+3 | 8 |  |
|  | **#5** | 4+2+2+2 | 3+3+3+3 | 2+2+1+1 | 9+7+6+6 | 7 |  |
|  | **#6** | 2+2+2+1 | 2+2+2+1 | 2+2+2+1 | 6+6+6+3 | 5.25 |  |
| **AAV-HBV 16W** | **#1** | 4+4+4+4 | 3+3+3+3 | 4+4+4+4 | 11+11+11+11 | 11 |  |
|  | **#2** | 4+4+4+4 | 4+4+3+3 | 4+4+4+4 | 12+12+11+11 | 11.5 |  |
|  | **#3** | 4+4+4+4 | 4+4+4+4 | 4+4+4+4 | 12+12+12+12 | 12 |  |
|  | **#4** | 4+4+4+4 | 4+4+3+3 | 4+4+4+4 | 12+12+11+11 | 11.5 |  |
|  | **#5** | 4+4+4+3 | 3+3+3+3 | 3+3+3+3 | 10+10+10+9 | 9.75 |  |
|  | **#6** | 4+4+4+3 | 4+4+4+3 | 4+4+4+3 | 12+12+12+9 | 11.25 |  |
| **Control** | **#1** | 3+3+3+3 | 4+4+4+4 | 3+2+2+2 | 10+9+9+9 | 9.75 |  |
|  | **#2** | 4+4+4+3 | 4+4+4+4 | 4+4+4+4 | 12+12+12+11 | 11.75 |  |
|  | **#3** | 4+3+2+2 | 4+3+2+2 | 4+3+2+2 | 12+9+6+6 | 8.25 |  |
|  | **#4** | 4+4+3+3 | 4+4+3+3 | 4+3+3+3 | 12+11+9+9 | 10.25 |  |
|  | **#5** | 4+4+4+4 | 4+4+4+4 | 4+4+4+4 | 12+12+12+12 | 12 |  |
|  | **#6** | 4+2+2+1 | 4+3+2+2 | 4+3+3+2 | 12+8+7+5 | 8 |  |

**Table S2. The gene-specific primers (5’ to 3’) used for RT-PCR for cytokines**

| **Genes** | **Forward** | **Reverse** |
| --- | --- | --- |
| **Hamster IFN-γ** | TGTTGCTCTGCCTCACTCAGG | AAGACGAGGTCCCCTCCATTC |
| **Hamster IL-6** | AGACAAAGCCAGAGTCATT | TCGGTATGCTAAGGCACAG |
| **Hamster IL-10** | GGTTGCCAAACCTTATCAGAAATG | TTCACCTGTTCCACAGCCTTG |
| **Hamster TNF-α** | TGAGCCATCGTGCCAATG | AGCCCGTCTGCTGGTATCAC |
| **Hamster γ-actin** | ACAGAGAGAAGATGACGCAGATAATG | GCCTGA ATGGCCACGTACA |
